# Recent Advances in Anodes for Microbial Fuel Cells: An Overview

**DOI:** 10.3390/ma13092078

**Published:** 2020-05-01

**Authors:** Asim Ali Yaqoob, Mohamad Nasir Mohamad Ibrahim, Mohd Rafatullah, Yong Shen Chua, Akil Ahmad, Khalid Umar

**Affiliations:** 1 School of Chemical Sciences, Universiti Sains Malaysia, Penang 11800, Malaysia; asim.yaqoob@student.usm.my (A.A.Y.); yschua@usm.my (Y.S.C.); khalidumar4@gmail.com (K.U.); 2 School of Industrial Technology, Universiti Sains Malaysia, Penang 11800, Malaysia; akilahmad@usm.my

**Keywords:** microbial fuel cell, anode material, anode fabrication source, wastewater treatment, energy production, anode challenge

## Abstract

The recycling and treatment of wastewater using microbial fuel cells (MFCs) has been attracting significant attention as a way to control energy crises and water pollution simultaneously. Despite all efforts, MFCs are unable to produce high energy or efficiently treat pollutants due to several issues, one being the anode’s material. The anode is one of the most important parts of an MFC. Recently, different types of anode materials have been developed to improve the removal rate of pollutants and the efficiency of energy production. In MFCs, carbon-based materials have been employed as the most commonly preferred anode material. An extensive range of potentials are presently available for use in the fabrication of anode materials and can considerably minimize the current challenges, such as the need for high quality materials and their costs. The fabrication of an anode using biomass waste is an ideal approach to address the present issues and increase the working efficiency of MFCs. Furthermore, the current challenges and future perspectives of anode materials are briefly discussed.

## 1. Introduction

Improper wastewater treatment is a serious threat to the maintenance of a green environment for human beings. Water pollution, water shortages, and energy crises are creating a serious and alarming situation for human lives worldwide [1,2]. The microbial fuel cell (MFC) approach accurately counters the above-mentioned crises. In the modern era, the MFC approach has received much interest because of its unique methods to achieve energy and wastewater treatment [3]. MFC is an emerging and significant approach that decontaminates toxic pollutants and simultaneously converts chemical energy into electrical energy by using bacteria that serve as catalysts [4,5]. Significant achievements in wastewater treatment and reasonable power density have been reported. However, MFCs have not yet been implemented at a commercial scale due to their low energy production and low removal efficiency. There are several reasons for low energy production or removal efficiency, such as low-quality materials being used as anodes or material cost issues. The anode is one of the most important parts of an MFC and provides the necessary surface area for bacterial growth. Further, these bacteria generate electrons and protons and transfer to the anode. However, designing anode materials remains a challenge for MFC performance [6]. Recently, interest in anode configurations, materials, and design has progressively increased for high-performance MFCs. Anode materials must have a few basic properties to meet high-performance requirements, such as good biocompatibility, high conductivity, high chemical stability, good thermal and mechanical stability, and a large surface area. A large number of anode materials are widely used in MFCs, but these materials have some drawbacks that make this approach unsuitable for commercial applications [7,8]. The previous literature showed that modification of the anode to achieve a high surface, good electron transferability, and bacterial adhesion has become a new research interest in the field of MFCs. 

**Figure 1:**
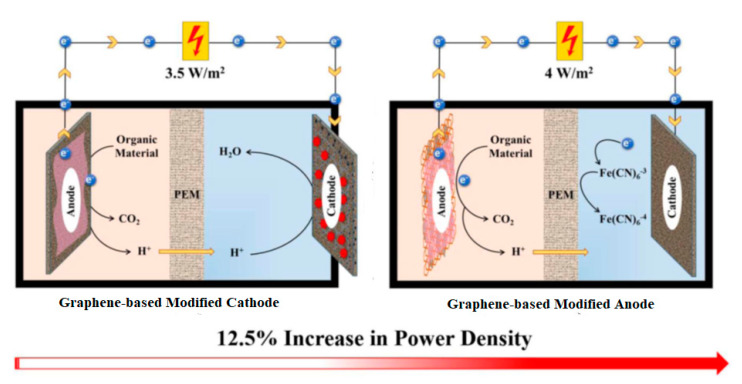
Superiority of the graphene-based modified anode (**right**) compared to the graphene-based modified cathode (**left**) (reproduced from Reference [9] with Elsevier’s permission).

The fabrication of the anode is a crucial step because the anode is responsible for providing a high surface area to the bacteria to produce electricity. To date, however, no material has been offered the opportunity to be employed at a large scale. In MFCs, both electrodes (anode and cathode) play a vital role, but only the anode is responsible for bacterial growth, removal rate, electron generation, and the transformation to a cathode. According to El Mekawy et al. [9], the anode is a very important part of the MFC approach. The authors studied the applications of graphene derivatives as electrodes and compared their performance in terms of the anode and cathode electrode. From this study, the authors concluded that a graphene derivative-based anode is superior to a cathode in terms of energy production, as shown in Figure 1. However, carbon-based materials are the most significant and emerging material for use as an anode [10]. This study also provides a new research direction by reducing the costs of the materials. Among carbon-based materials, the most efficient materials are graphene derivatives for the anode. Today, it is possible to synthesize graphene oxide by using domestic and industrial waste materials. Similarly, many metals and conducting polymers that exhibit good performance in the form of composites exist. These composite materials are also considered a better option to enhance the performance of electrodes, especially for anode electrode synthesis. Despite all this progress, unmodified graphene derivative materials are not preferable due to their toxic effects on bacteria. On the other hand, unmodified metal derivatives also suffer from corrosion issues in MFCs [11,12]. Therefore, graphene derivatives with metal/metal oxide nanoparticles or conductive polymer-based composite materials are used as anodes to enhance the working efficiency of MFCs. The large surface area and excellent conductivity of composite materials offer a favourable environment for stable relationships between bacteria and the anode material. Thus, anode (rather than cathode) modification is a necessary step when using graphene derivatives to achieve better outcomes in MFC operations. In this review article, we summarize the different types of anode materials along with their electricity generation, inoculation sources, surface modifications, electrode sizes, and design. Different possible fabrication sources are discussed to investigate the importance of waste biomass as a new research direction. Finally, the effects of the anode on wastewater treatment and electricity generation are discussed, and some emerging challenges and future prospects are outlined.

## 2. Electrode Materials

The electrode materials were studied to optimise the removal efficiency of pollutants and energy production. The electrode material should be highly suitable in terms of its mechanical strength, chemical stability, biocompatibility, and electrical conductivity. The efficiency of anode and cathode materials is summarized below. 

### 2.1. Anode Materials

There are many materials that can be used to fabricate a perfect anode for MFCs which depend upon a larger surface area by increasing extracellular electron transfer efficiency through a biofilm. However, anodic materials are also essential because they help to enhance the metabolic rates for anaerobic microorganisms to oxidize organic waste [13,14,15]. It is already known that bacteria (concentration and types) have a great influence on power density in MFCs. Thus, the selection of materials for the anode must be appropriate. High performance anode materials are a significant aspect for use in MFCs. However, several studies have been focused on the improvement of anode materials through diverse modification approaches. Therefore, it is crucial to find more resources for anode material preparation. The most commonly used sources are carbon-based, metal or metal oxides, conducting polymers, or composite materials, which are considered to be potential materials for anode preparation with significant value. 

#### 2.1.1. Carbon-Based Materials

Recently, carbon-based materials have been commonly used to fabricate electrodes due to their high chemical and mechanical stability, cost effectiveness, high conductivity, good biocompatibility, and good electron transfer kinetics. Based on our extensive literature review, some common carbon-based material, such as carbon felt, rod, fibre, cloth, mesh, paper, activated carbon cloth, glassy carbon, brushes, reticulated vitreous carbon and graphite (block, felt, 3D graphite, graphite oxide, and granular graphite) have been studied. One of the recently emerging materials known as graphene has gained much interest for use in MFCs as an electrode material. 

In conventional carbon-based materials, carbon rods, paper, felt, cloth, brushes, meshes, etc. are the most commonly used materials in MFCs. Wang et al. [16] examined that a carbon mesh is slightly less expensive than other carbon forms and also offers better current density. However, the modification of carbon meshes by treating them with ammonia (or other types of) gas to improve their performance can offer acceptable results. Therefore, there is currently no untreated material that offers better power density. Single carbon granules’ performance as capacitive bioanodes was studied by Borsje et al. [17]. The outcomes were based on charge storage performance and current production through a single carbon granule. The activated carbon granule can store charges in the form of an electric double layer, which increases bioanode performance. Granular and activated graphite carbon granules have been employed to determine the unexploited outcomes of granular bioanodes. Single activated carbon-based granules generate 0.6 mA at −300 mV vs. Ag/AgCl anodes. The charge or discharge mechanism illustrates that capacitive granules generate 1.3–2 times the additional charge of graphite granules with lower superficial areas [18,19,20]. Similarly, Li et al. [21] studied granule activated carbon, which produced two-times greater energy than conventional carbon materials. This study concluded that granule-activated carbon could be an effective alternate source of anode preparation.

Carbon cloth/sheets provide a reasonable surface area for the growth of bacteria and show flexible characteristics. However, this material is also not suitable at larger scales due to its high cost [22]. The authors observed that activated carbon cloth has a high surface area and better adsorption ability in sulphide electrochemical oxidation at the anode for the removal of sulphide along with better current production. In a previous study, Wang et al. [23] prepared a doped carbon cloth that offered a high current efficiency of 2777.7 mW/m^2^. When carbon cloth was doped with nitrogen gas, it yielded high power efficiency, which might be useful for future studies.

Similarly, graphite is another form that is commonly used in MFCs as an electrode material. A crystalline carbon form with sp^2^ hybridization is known as graphite. Due to its high conductivity and better stability, graphite is a potential anode material in MFCs. To fabricate a plain anode, graphite sheets or plates, clothes, brushes, granules, etc. are effectively used [24,25,26]. Ter-Heijne et al. [27] observed that rough graphite showed higher current density when used as an electrode in MFCs instead of flat graphite. However, they have a high cost and a relatively lower surface area, making this material unsuitable to use at a commercial level of energy production. Lowy et al. [28] reported the performance of a graphite brush and considered graphite brushes as a model electrode material to use as an anode in MFCs to enhance energy production and remove toxic pollutants. Later, Yazdi et al. [29] used two graphite brushes with different diameters (2.5 and 5.0 cm^2^) with high surface areas of 18,200 m^2^/m^3^ and 7170 m^2^/m^3^. These small brushes with a 2.5 cm^2^ diameter showed the highest current power of 2400 mW/m^2^ with 60% columbic efficiency. This proved that a high surface area of the anode materials plays an important role in high removal efficiency and current generation because it enhances bacterial growth on the anode’s surface. Similarly, Zhang et al. [30] studied a graphite brush with a 5 cm^2^ diameter and observed a 1430 mW/m^2^ power density, which is greater than that when using carbon paper as an anode electrode. This means that smaller sized brushes can produce greater energy output than larger brushes. Similarly, graphite’s reported power density was 1771 mW/m^2^ for an electrode during the waste water treatment of the Cassava mill [31,32,33]. Carbon-based material are also used in a packing form to enhance the surface area for bacteria [34]. Graphite carbon is also used in its packing configuration due to its larger specific surface area. There are several strategies to improve the performance of graphitic materials as anodes, such as graphite doping with metal/metal oxides. In another study, Yasri et al. [35] doped graphite material with calcium sulphide to prepare an efficient anode material to increase bacterial contact with the anode and reduce electrical potential. The authors also studied graphite doped iron-based compounds and observed that they showed better performance compared to previously synthesised anode materials.

A newly emerging carbon allotrope known as graphene (present in 2D hexagonal lattice) has received much attention in the modern era. Graphene is considered to be an effective potential material for anode preparation due to its advanced properties of high conductivity and good thermal and mechanical strength. Graphene has nonlinear and superior diamagnetism compared to graphite materials. However, presently, graphene and its derivatives are still under research for use as anodes in MFCs [36]. There are various reported methods to synthesize graphene. Commercially available graphene is quite expensive, but using waste materials to fabricate graphene is less expensive [37,38,39,40]. Graphene as an anode provides high scale working efficiency for MFCs due to its high energy production compared to other conventional carbons. All aforementioned conventional carbon-based electrodes offer less efficiency than graphene-based electrodes as anodes due to the high-performance properties of graphene [41]. Graphene exerts non-toxic effects on bacterial growth during MFC operations. Thus, its modification or combination with other materials, such as metal or conductive polymers, can minimize the toxicity effect of other materials, such as copper [42]. Modified carbon allotropes could yield a revolution in the fields of energy and wastewater treatment.

#### 2.1.2. Natural Biomass Materials for the Anode

**Figure 2:**
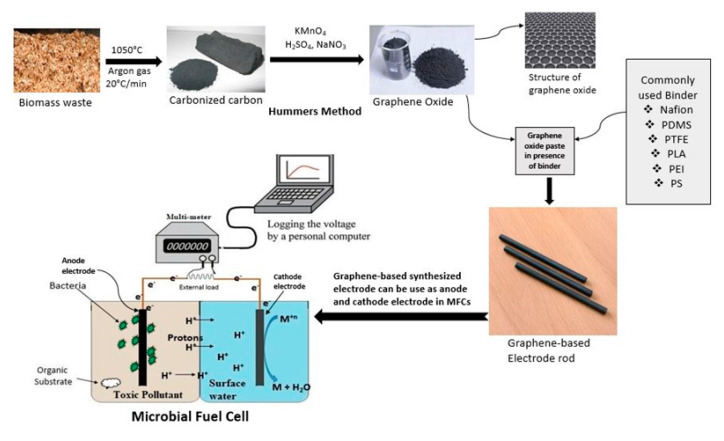
Systematic presentation of electrode fabrication for MFCs by using biomass waste.

Electrode materials differ in their chemical, physical, and biological properties. Electrode materials must be biologically compactable with bacterial species so they can influence microorganism attachment, the transfer of electrons, and the rate of reaction on the surface of electrodes, as well as the resistance of electrodes [43]. However, the selection of electrode materials and the preparation of electrodes have become interesting and emerging research directions in recent years. Using waste materials for MFCs has seen little application in the fabrication of electrodes. Much time is required to change waste materials into valuable materials to develop electrodes, but this process is very effective compared to commercial materials in terms of several parameters [44]. Cheng et al. [45] studied a prepared reduced graphene (rGO) composite for anodes by using waste material to achieve more effective results in terms of energy generation and wastewater treatment through MFCs. Green rGO was effectively prepared using dried eucalyptus leaves, which are a waste material. Later, nanocomposites of rGO/gold nanoparticles were successfully prepared and biocompatible electrodes were fabricated through a layer-by-layer assembly coating technique. This modified electrode achieved a 69.4 A/m^3^ current density and 33.7 W/m^3^ power densities. The prepared nanocomposite-based electrode provides greater surface roughness for superior bacterial colonization. The presence of gold nanoparticles in the composite provides high electroactive sites and enables the transfer of electrons from electricigens to an anodic site. Singh et al. [46] derived carbon nanoparticles from candle soot and used this waste source to prepare an effective electrode for MFCs. The candle soot was coated on the surface of a stainless-steel disk, and this disk allowed the carbon nanoparticles to be employed directly as electrodes. The physical, electrical, and chemical characterization results revealed that the electrode materials had good electrochemical and mechanical stability with hierarchically porous characteristics. The polarization outcomes revealed 0.68 ± 0.03 V as the highest circuit potential, 7135 ± 110 mA/m^2^ as the current density, and 1650 ± 50 mW/m^2^ power in the dual chamber of the MFCs. The preparation of candle soot-based carbon nanoparticle electrodes is recyclable, cost-effective, scalable, and reliable. Similarly, Bose et al. [47] used biomass to fabricate an activated carbon-based cathode for bioenergy production through MFCs. This was a unique method to generate electricity and water treatment without any environmentally hazardous effects. Usually, platinum is used as a catalyst for oxygen reduction at the cathode site. The authors also evaluated the performance of activated carbon derived from sugarcane waste in terms of its stability, functionality, and cost. This waste material followed the carbonization process at different temperatures (300, 400, 500 °C) at 60 min intervals. The results showed a 0.40 mA/m^2^ current density and a 110 ± 6.58 mW/m^2^ power density. Electrodes derived from several biomass resources are also a promising alternative to treat environmental pollutants and simultaneously generate electricity. According to our knowledge, there have been few publications on biomass-based anodes in MFCs. The reusability concept of waste biomass materials is a promising alternative to increase the working efficiency of MFCs without any high expenditures. The most popularly emerging and promising material for electrodes is graphene and its derivatives, which can be easily prepared via many methods, such as the scotch tape method, epitaxial growth, reduction of CO, chemical vapour deposition, electrochemical synthesis, confined self-assembly, exfoliation, arc discharge, and Hummer’s method. The most important method is Hummer’s method due to its significant advantages over other methods. For example, it is an eco-friendly method, no harmful gases are generated during preparation, the product has an organized structure, and a larger amount of product is provided. Recently, Hung et al. [48] used a renewable waste coffee-based anode to enhance the power density in MFCs. The authors converted waste materials into valuable carbonized materials and applied them as an anode in an MFC by reducing waste from the environment. The achieved power density was 3800 mW/m^2^, which is much higher than that of conventional materials. Several types of waste material are available in our surroundings and cause serious hazards. Therefore, a positive approach is to convert biomass waste materials into valuable materials. However, in Hummer’s method, various waste materials (biomass, domestic, and commercial) are carbonized under the influence of argon gas at 1050 °C to obtain fine carbonized powder materials. The obtained graphitic powder is treated with the oxidizing agent KMnO_4_/ H_2_O_2_ in order to obtain graphene oxide. The synthesized graphene oxide can be further functionalized with polymeric binders such as nafion, polyethyleneimine, and polylactic acid to fabricate the graphene oxide material into an anode electrode shape [49]. The prepared graphene oxide material can be used as an anode or a cathode, but the anode is superior, as mentioned earlier. This kind of fabricated material can enhance the performance and reduce the cost of the material, making it effective at a larger scale. Using low cost synthesised composite materials coupled with metal oxides such as TiO_2_/GO, ZnO/GO, and CuO/GO is an ideal approach to address several current challenges. The anodes prepared by using natural biomass resources in the last few years are summarized in Table 1. The systematic synthesis paths of the anode electrodes for MFCs are shown in Figure 2.

#### 2.1.3. Metal and Metal Oxide-Based Materials

Other commonly used materials for the fabrication of anode electrodes are metal and metal oxides, but corrosion limits the use of metal-based electrodes, especially in the case of anode for MFCs. Metals are generally more conductive than carbon-based materials because metals can facilitate effective electron flow [58]. Metals contain unique properties, but not all metals are widely used for electrode fabrication due to the process’s noncorrosive requirements. Moreover, some metals do not facilitate bacterial adhesion. For example, non-corrosive stainless-steel materials do not offer high power density compared to other carbon-based materials like graphene and graphite. Generally, the smooth surfaces of metals do not facilitate the adhesion of bacteria. Some non-corrosive materials, such as stainless steel, fail to achieve higher power densities than carbon-based materials. The power density of stainless steel was found to be 23 mW/m^2^ at the anode chamber [59]. A stainless steel grid used as an anode generated a higher current density than a plain graphite electrode [60,61]. However, some metals are much better for use as anode materials, such as gold, silver, titanium, and platinum. Noble metal-based anode electrodes help to decrease the internal resistance in MFCs, but their higher cost and poor bacterial adhesion prevent their extensive integration during the operation of MFCs [62,63]. Titanium and platinum are mostly used as catalysts to enhance the performance of anode electrodes [64]. However, it is not easy to commercialize pure metal-based anodes in MFCs at a large scale due to their high cost and limitations. The catalytic activity of metal oxide nanoparticles and non-noble metal is equivalent to that of valuable metals, which can significantly decrease resistance and improve the attachment of bacteria on a surface. Nanometallic particles also provide a great opportunity to minimize the effect of toxicity to bacterial cells [65,66]. These problems can be reduced by modifying metal/metal oxide nanoparticles (ZnO, Ag, TiO_2,_ etc.) with other materials, such as carbon-based or conductive polymers. 

#### 2.1.4. Conductive Polymer-Based Composite Material

**Figure 3:**
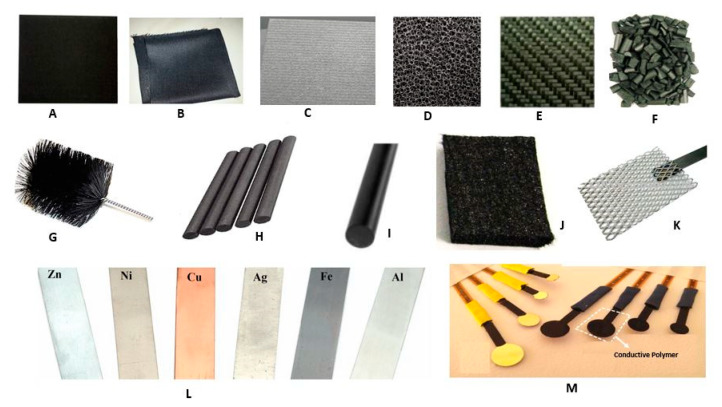
List of commonly used electrodes: (**A**) carbon paper, (**B**) carbon cloth, (**C**) carbon fibre, (**D**) reticulated vitrified carbon, (**E**) carbon mesh, (**F**) graphitic granular, (**G**) carbon brushes, (**H**) graphite rod, (**I**) polycrystalline graphite, (**J**) carbon felt, (**K**) platinum mesh, (**L**) different metal electrode strips, (**M**) conductive polymer-based strips.

Conductive polymers such as polyaniline, polypyrrole, polythiophene, poly-co-o-aminophenol, and many others can serve as anode materials due to their high electronic conductivity characteristics [67,68,69]. Conductive polymers offer excellent results through modification with other carbon-based materials. For example, carbon cloth modification with polyaniline showed excellent power generation compared to unmodified materials [70]. Graphite felt and polyaniline composites served as anodes and revealed a 2.9 W/m^3^ power density, which was much better than the results for an unmodified anode. Moreover, this composite offered a much high surface area for bacterial growth [71]. Another conductive polymer called polypyrrole is one of the good materials that showed a 452 mW/m^2^ power density with the modification of carbon paper [72]. According to the literature, polypyrrole can penetrate into the cell membranes of bacteria and carry the electron through the metabolic pathway [73]. Thus, conductive polymer composites with other materials, such as carbon-based materials, metals, and their derivatives, could greatly enhance the working efficiency of electrodes. For example, Dumitru et al. [74] studied polypyrrole and polyaniline with carbon nanotubes (CNTs) in the form of a nanocomposite-based anode. The obtained power density was, for the CNT/polyaniline (202.3 mW/m^2^) and CNT/polypyrrole nanocomposites (167.8 mW/m^2^), higher than that of the unmodified CNTs (145.2 mW/m^2^). CNTs and the conducting polymer nanocomposites offer reasonable performance, particularly in electrochemical applications, due to their synergistic influence [75]. Different metals, such as Ag, Zn, TiO_2_, Cu, and a ZnO composite including conductive polymers (especially polyaniline and polycarbazole) could offer a great opportunity to upgrade MFC performance [76,77,78]. Unfortunately, very little effort has been made in this direction to prepare polymeric composite-based electrodes in MFCs. Some commonly used electrodes, such as carbon-based, metal-based, and conductive polymer-based electrodes, are shown in Figure 3.

### 2.2. Cathode Materials

The cathode material has the most significant impact on the working performance of MFCs after the anode. Currently, the most commonly used materials for cathodes are carbon-based. Material efficiency, electrode size, and modelling are also problematic challenges for cathodes [79,80,81,82]. Most of the reported materials, as stated in the anode section, can serve as a cathode. Commonly, substrate reduction reactions occur at the cathode chamber due to deprived catalyst activities limiting MFC performance [83,84,85,86]. Usually, cathodes are categorized into two major forms: air-cathodes and aqueous air-cathodes with or without catalysts. The major difference between these configurations is the catalyst. The most commonly used catalysts are platinum and titanium to enhance performance. Another difference is that an air cathode is always exposed directly to oxygen [87]. This configuration has received considerable attention because of its functional simplicity, suitable electrode design, and lack of aeration. An air cathode offers a high possibility to enhance the power output through MFCs [88,89]. In aqueous air cathodes, conductive materials are used to make electrodes, such as platinum meshes, carbon felt, carbon fibre, and carbon cloth, attached to the catalyst layer that is already present in the aqueous regions with low oxygen contact [90]. The carbon cloth is considered to be the most suitable conductive material for use as an air cathode. Binder materials are used to prepare air cathodes to fix the catalysts (platinum, titanium, or copper) used on the electrodes [91,92,93]. The most attractive binders are poly(tetrafluoroethylene) and perfluorosulfonic acid (nafion). Zhang et al. [94] studied activated carbon and carbon cloth as cathode electrodes by using poly(tetrafluoroethylene) as a binder to compare the performance of both materials. The results revealed that the activated carbon offers higher power density compared to carbon cloth (1220 mW/m^2^ and 1060 mW/m^2^) in the presence of Pt as a catalyst. Therefore, activated carbon could be a good alternative for the preparation of cathode materials.

Zhao et al. [95] used a Pt catalyst with carbon cloth for the preparation of a cathode. Based on the results, this catalyst produced 1.2 W/m^3^ power efficiency. Generally, at lower temperatures, Cu is a more selective catalyst compared to Pt because Cu can maintain selectivity better than Pt. Otherwise, Pt is a more excellent and well-established catalyst than others under room conditions. Wang et al. [96] observed carbon paper in the presence of a Pt catalyst to yield 457.8 ± 15.2 mW/m^2^ power efficiency. Thus, the materials used for anodes and cathodes can serve as catalysts to catalyse oxygen reduction. Platinum and gold are considered potential catalysts due to their low overpotential, but their high cost makes them unsuitable [97,98,99]. To solve this issue, primary transition metals offer a better alternate, as they are comparatively inexpensive, offer high stability, and do not disturb the microbial environment within the cell. Some composite materials, such as tungsten carbide or molybdenum, also offer excellent results, while stainless steel and nickel alloys provide the best performance [100]. Nanocomposites, however, are less expensive and offer a great opportunity to improve the working efficiency of MFCs (for example, palladium nanoparticles and Ni nanomaterials [101]). Nanomaterials have a greater surface area, higher mechanical and thermal stability, and superior electrochemical activity compared to other materials [102,103]. The new trend involves modification of the electrode with other materials to enhance the oxygen reduction reaction. According to the literature, there is a crucial need to study novel materials to enhance the quality of electrodes, especially anodes. The use of high-quality materials, such as graphene and its derivatives featuring metal oxide as a composite material, for anodes could precipitate a great change in the field of MFCs. The most suitable composites are GO/ZnO, GO/TiO_2_, and GO/Ag, which have a great impact on power generation. Both graphene oxide and metal oxide are easily synthesized using waste materials to reduce the cost of anodes. Furthermore, the various types of traditional carbon-based materials, metal/metal oxides, and conductive polymers that can be used as anodes and cathodes in MFCs are reported in Table 2.

## 3. Effects of Anodes in MFCs

**Figure 4:**
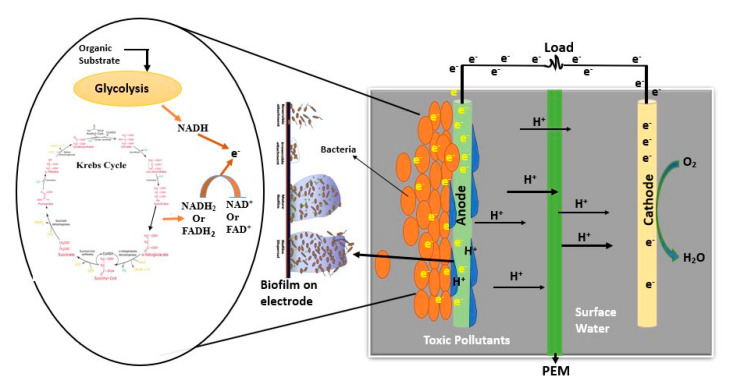
Performance of anode electrode in MFCs.

The anode is a significant element that is also responsible for the removal of toxic pollutants and the generation of electricity in the presence of a biocatalyst during MFC operations. Bacteria are associated with the anode surface to generate electrons and protons during their respiration. The electrode provides a great enough surface to allow bacteria to grow and oxidize, as briefly described in Figure 4. The anode’s performance provides MFCs with great electric output, the bioremediation of wastewater, and compactable economic attributes.

### 3.1. Effect of Anode on Removal of Pollutants

The bioremediation of wastewater is considered to be a very efficient potential application of MFCs. Many conventional methods have been reported for wastewater treatment, but they are all associated with some drawbacks, such as high costs, a risk of self-toxicity, being difficult to operate, and remaining unstable for environmental safety [35]. The MFC approach is a potential technique for the successful bioremediation of several types of wastewater, such as petrochemical industrial wastewater, swine wastewater, seafood processing wastewater, livestock waste, vegetable and food-processing waste, slaughter house wastewater, corn stover waste, dairy wastewater, surgical cotton industry waste, and cassava mill wastewater [139]. In the case of organic pollutants, the oxidation of organic substances produces protons and electrons in the anode chamber with the help of exoelectrogens and degrades the toxic organic pollutants in water [140,141]. These protons are transferred to the cathode directly or through a membrane sources, and the electrons moved through the outer circuit. This process depends upon the working efficiency of the electrodes. The electrodes provide a surface area to bacteria for their growth and respiration processes, which facilitate the transfer of electrons and protons from the bacteria to the anode and then transfer them to the cathode. Zhang et al. [142] studied the reduction of Cr (VI) and V(V) with the generation of electricity through double chamber microbial fuel cells by using vanadium-based wastewater that served as a cathode-based electron acceptor. Cr (VI) and V(V) are two major metals present in vanadium-based wastewater with great toxicity and a large quantity. The reduction efficiency of Cr (VI) and V(V) after 10 days of operation using carbon fibre felt as the anode and cathode electrode was 75.4% ± 1.9% and 67.9% ± 3.1%, with 970.2 ± 20.6 mW/m^2^ power density. 

Later, Qiu et al. [143] also studied the reduction of vanadium by using a biocathode in MFC and achieved a 60% removal rate in the presence of *Dysgonomonas* and *Klebsiella* (biocatalyst). The achieved power density was 529 ± 12 mW/m^2^ after a seven-day operation of MFCs with a 200 mg/L initial concentration of anaerobic sludge. The carbon fibre felt (40 × 40 × 10 mm^3^) was used for both the anode and cathode electrodes, which were coupled through external resistance (100 Ω). Jiang et al. [144] studied oil sand process-based wastewater to determine the capability of MFCs to produce energy along with the treatment of oil sand tailings. After 35 days of continuous operation, the observed highest voltage was 0.726 V in the presence of a 1200 Ω resistance loaded. Later, after 70 days, the maximum power density reached around 392 mW/m^2^. With constant energy production, the MFCs removed some heavy metals from the oil sand process-based wastewater, and the observed efficiency was 66.9% (Cu), 4.9% (Cr), and 32.5% (Pb). The removal efficiency was quite low due to the presence of carbon cloth as the anode and cathode. However, the carbon fibre felt offered a higher removal efficiency than the carbon cloth for several reasons, but the quality of the anode and the surface area provided to the bacteria played a significant role. This has a direct effect on removal efficiency because bacteria require an active surface area for respiration to degrade the pollutants. Therefore, to enhance the quality of the anode, Habibul et al. [145] used a graphite-based anode to study the electro kinetic bioremediation of heavy metals, especially Pb and Cd from polluted soils. After 108 and 143 days of operation, the achieved removal efficiency was 44.1% and 31%, respectively. The power density of Cd and Pb was 7.5 mW/cm^2^ and 3.6 mW/cm^2^, respectively. The rate of voltage generated by the bacterial culture was comparatively low due to the lower efficiency of the electrodes for the highly toxic metals, Pb and Cd. However, very few studies are available on the degradation of highly toxic metals such as Pb, Hg, and Cd. Bacteria require healthy and high-quality anode materials to degrade toxic metals from the water system. Similarly, the authors used anode materials in MFCs to decolorize the organic dyes which produced serious hazards in the environment. Fang et al. [146] investigated the potential of MFCs by employing activated carbon as an anode electrode and a stainless-steel mesh as the cathode to treat the azo dye from concentrated anaerobic sludge. The rate of decolorization was 95.6%, and the power density was 0.852 W/m^3^. The rate of decolorization was high because the activated carbon was working as the anode. Kawale et al. [147] used an unpolished graphitic rode as the anode to decolorize the methyl orange from anaerobic sludge. The rate of decolorization was 73.4% with a maximum current density of 0.13 ± 0.03 A/m^2^ through double chamber MFCs. The effect of the electrode was very substantial for both decolorization and energy production. However, some studies carried out the removal of organic pollutants via MFCs with different anode materials. Kabutey et al. [148] studied the removal of organic pollutants and energy production from an urban river sediment by employing a macrophyte cathode sediment microbial fuel cell. Carbon fibre brushes were present as both the anode and cathode electrodes in operation and achieved a 28.2% removal efficiency. The removal efficiency was low because the produced bacteria *Euryarchaeota* and *Proteobacteria* could not remove the phosphorus due to its acidic nature, so a low-quality electrode was employed. Later, Marks et al. [149] studied the performance of MFCs by using an anoxic environment and achieved 22% nitrate removal efficiency from anaerobic sludge. Graphite plates were used as anode and cathode electrodes in this study. According to an extensive literature review, the authors concluded that there are several type of anode materials used under different conditions because several factors are responsible for MFC performance. One of the most important factors of an anode is to provide a large enough surface area to bacteria for their respiration and to help them carry the electrons from the bacteria to the cathode by using the outer circuit. Therefore, it is expected that using a high-quality anode would obtain better results without any high environmental requirements for operation. This high-quality material could address many challenges that create disturbance during operation, such as long-term stability. We could make this anode more efficient by using highly conductive and high surface area materials such as graphene and its derivatives or composite materials with metal/metal oxides to avoid the metal corrosion issue. Therefore, in terms of wastewater treatment, the anode must be efficient and unique to achieve better results.

### 3.2. Effect of the Anode on Energy Production

MFCs have created a revolution in the field of environmental pollutants and their safe removal. MFCs use bacteria as exoelectrogens to generate electricity from different organic waste substances [150,151]. The energy production of the anode or cathode electrode has been enhanced over the years from less than 1 mW/m^2^ to 1 to 4 W/m^2^ due to several developments, such as design improvements, introducing single chamber MFCs, using 3D electrode materials, and the development of improved and highly-valuable anodes [117]. In the beginning, many materials and working parameters were frequently reformed, making it difficult to precisely identify the working factors that helped improve the current generation compared to conventional methods. Due to the progress of this system, more attention is now needed to generate a higher output of electricity [152,153]. The electrode is directly associated with power generation. As the quality and conductivity of the electrode increases, so too does the generation of energy. Furthermore, Wang et al. [81] demonstrated the efficiency of carbon felt as an anode in the presence of platinum as a catalyst, but the power output was quite low at 457.8 ± 15.2 mW/m^2^. Similarly, Zhang et al. [142] used integrated adsorption technology to remove the chromium from anaerobic digestion sludge and achieved good current power of 343 mV. Carbon felt was used as an anode in an MFC system to achieve 343 mV. Liu et al. [154] studied MFC performance by utilizing carbon brushes and cloth as the anode and cathode electrodes in the presence of iron–nitrogen/activated carbon as a catalyst and achieved a 1092 mW/m^2^ energy output by utilizing synthetic solutions. Therefore, to upgrade the material, Santoro et al. [116] sought to produce a high energy output by using graphite brushes as anodes in the presence of platinum catalyst SMFCs. The achieved power density was 1280 mW/m^2^ after using native wastewater as an inoculum source. Energy production depends on the performance of the electrodes. Graphite-based materials, for example, have higher surface areas and higher conductive efficiency than carbon felt. Therefore, the results of graphite-based materials are three times greater than those of carbon felt material. Nguyen et al. [155] further used the modification approach to increase the quality of the anode’s material. The authors used *E. coli* as a biocatalyst to produce electricity with the help of activated doped carbon paper containing a carbon nanotube composite. The energy output was 3.9 μW/cm^2^ after operating in a double chamber MFC. Recently, Zhang et al. [156] reported the high electrochemical performance of MFCs functionalized with carbonaceous allotrope graphene oxide as an anode. The graphene oxide enhanced the electron transfer and generated higher energy output than the other simple carbon-based materials. Thus, graphene is the most promising material for MFCs as an electrode to generate electricity.

However, in modern research, the scientific community uses natural resources for anode preparation because such resources are cost effective and high-performance materials compared to existing commercial materials. Yang et al. [157] observed the direct generation of electricity from banana peels and subaqueous wetland sediments used as an anaerobic sludge (an inoculum source) for MFC operation. The highest current densities observed were 78.2 mA/m^2^ and 91.3 mA/m^2^ for the collected banana peel material and subaqueous wetland sediments using carbon felt as electrodes in both chambers of the MFC. Several natural sources and their preparation methods are briefly summarized in Section 2.1.2, and their energy generation performance is summarized in Table 1. Therefore, using natural materials as anodes is a functional approach for addressing the current issues and to synthesize high-quality material for anodes, such as GO and their composites coupled with metal oxides. GO composites coupled with metal oxides can improve the properties of the anode. ZnO/GO, TiO_2_/GO, and CuO/GO based anodes are extensively used to achieve high electricity performance in MFCs. Over the past decade, several research groups from around the world have carried out research on using anode electrode materials to increase energy production and enhance the rate of pollutant removal, especially metals and organic dye pollutants (which are a serious threat to the environment), using MFCs (Table 3).

## 4. Challenges and Future Perspectives

Despite all the developments in MFCs, the scientific community still faces many challenges and problems in terms of electricity generation and water pollutant treatment. There is undeniably rapid development in making MFCs more prolific. Moreover, MFC reactors of different designs have been introduced, such as single and double chamber, H-shape, membrane-less, and tubular MFCs [181,182]. The main goal of these developments is to achieve the practical implementation of MFCs for wastewater at a commercial level. One of the main components in MFCs is the anode, which is also responsible to some extent for their economic and functional stability. There are several challenges associated with anodes which limit the usage of MFCs at an industrial scale:

1. The anode materials are very important for the economic stability of MFCs. Therefore, reducing the costs for anode materials is a serious problem for practical implementations in MFC applications. To solve this issue, we should focus on waste materials and change them into a carbonized form that can be further used as anode material in several forms, such as rods, brushes, and plates. Waste materials are a good resource for making carbon-based materials. However, another method is the development of composites with metals and using polymers to make them more efficient at a low cost. The selection of materials is also a major issue for MFC operation because most researchers use conventional materials, and very little work seems to have been done on highly conductive materials or composite materials [183].
2. During the development of an anode, the binder material is very important for fabricating the material in the required shape. The selection of binders is a very critical factor for a researcher because the binder serves as the binding agent in the material to make that material more cohesive and stable. It is desirable to find more suitable and cost-effective binders for anode electrodes. To the best of our knowledge, there is no comprehensive review article that reports on using binders for electrode fabrication.
3. The electrode’s size and design are very important aspects in the fabrication of anodes. The sufficient electrode spacing and surface area of the electrode are responsible for bacterial growth and electron transformation from the anode to the cathode in MFCs [184].
4. The modification of anode electrodes has produced major improvements in MFCs regarding power generation and the bioremediation of wastewater. However, the relevant mechanisms and proper guidelines remain unclear. Researchers must explore a more proper mechanism so modifications can be made more efficiently.
5. Another problem is the long-term stability of anodes at the industrial level. Currently, most studies focus on energy output, but no one has yet published any guidelines or discussions about electrode stability over the long-term [185,186]. Stability is a major issue that limits MFC applications at an industrial scale. Thus, researchers should focus on finding an effective fabrication technique for electrodes while keeping in mind the stability factor for anodic materials. A highly stable binder like nafion or polysulfones can be employed to bind the graphene oxide material to make anode electrodes able to maintain their long-term stability.

## 5. Conclusions

This review summarized the effects of anodes in MFCs. We discussed several types of materials that serve as anode in MFCs, such as carbon-based materials, metal/metal oxide-based materials, conductive polymers, and composite-based materials. For the development of anode electrodes, the key area of progress is the attachment of bacteria and the development of biofilm. Significant efforts have been focused on increasing the surface area of anode materials to attain greater biofilm densities. There are many alternative proposed materials for use as anodes, as mentioned in this review article. There is still a major gap, however, in the development of potential materials for anodes. Highly absorbent and highly conductive materials like 3D graphene and metallic composites can be utilized as anode electrodes in MFCs. Anode materials must be very stable in wastewater during MFC operation over the long term. These properties make an anode more valuable at an industrial scale if it remains stable over a long period of time. Therefore, an anode material must possess a significant pore size to avoid clogging issues in the bioremediation of wastewater applications. Presently, material cost issues and the unclear mechanism of surface modification hinder the practical application of MFCs. Hence, inexpensive and accessible materials and effective methods for metallic or polymeric composites or carbon-based electrodes should be introduced to industries for MFC applications. In the future, essential efforts should be focused on testing the upscaling of resourceful anodes. Developing an anode/membrane combination as an excellent membrane-based electrode assembly for practical application is essential. However, the available efficiency of anodes is still not sufficient for use at a commercial scale. Further studies must focus on using waste material to fabricate anode electrodes and optimize current challenges.

## Figures and Tables

**Table 1:** List of anode electrodes synthesized using natural waste resource for MFCs.

Anode Materials	Surface Area of Electrodes (cm^2^)	Size of Electrodes (cm^2^)	Inoculum Source	Power Density (mW/m^2^)	Reference
Kenaf	2.5	0.23 × 1.52	Domestic sewage	-	[50]
Compressed milling residue	10.99	0.5 × 3.0	Anaerobic mix sludge	532	[51]
Bamboo charcoal	59.21	2.4 × 1.57	Anaerobic mix sludge	1652	[52]
Loofah sponge	10.99	0.5 × 3.0	Anaerobic sludge	701	[53]
Loofah sponge/PANI	10.99	0.5 × 3.0	Mix sludge	2590	[54]
Coconut shell/ sewage sludge	10.99	0.5 × 3.0	Mix sludge	1069	[55]
Barbed chestnut shell	91	2.7 × 2.7	Mix sludge	759	[56]
Silk cocoon	7	-	Mix sludge	5	[42]
Chestnut shells	125.65	0.3 × 66.4	Anaerobic mix sludge	850	[45]
Onion peels	7	1.0 × 2.0 × 0.5	Mix sludge	742	[57]
Coffee wastes	1	-	Domestic waste	3927	[48]

**Table 2:** List of the materials used as anodes in MFCs.

Type of Material	Electrodes		Size of Anode	Surface Area of Anode	Catalyst	Inoculum Source/ Bacteria	Power Density	Reference
	Anode	Cathode						
Carbon-based	Carbon cloth	Carbon cloth	2 cm × 2 cm	4 cm^2^	Without catalyst	*S. putrefaciens CN32*	679.7 mW/m^2^	[104]
Composites	rGO/SnO_2_/Carbon cloth composite	Pt rode	3 cm × 2 cm	6 cm^2^	Pt	*E. coli*	1624 mW/m^2^	[105]
Carbon-based	Graphene	Carbon cloth	*-*	4 cm^2^	Pt	*E. coli*	2850 mW/m^2^	[106]
Composites	r GO/PPy	Carbon paper	1 cm × 1.5 cm	*-*	Pt	*E. coli*	1068 mW/m^2^	[73]
Carbon-based	Graphene coating on Carbon cloth	Carbon cloth	1 cm × 2 cm	4 cm^2^	Pt	P. aeruginosa	52.5 mW/m^2^	[107]
Carbon-based	Graphene oxide modification with carbon paper	Carbon paper	5 × 3 cm^2^	-	-	Anaerobic Sludge	368 mW/m^2^	[108]
Composite	Polyaniline (PANI) networks onto graphene nanoribbons (GNRs)-coated on carbon paper (CP/GNRs/PANI)	Carbon paper	2 cm × 2 cm	4 cm^2^	Ti	*S. oneidensis MR-1*	856 mW/m^2^	[89]
Carbon-based	Graphene nanosheet coating on carbon paper	Carbon cloth	*-*	-	Pt	*S. oneidensis MR-1*	610 mW/m^2^	[91]
Composites	N-doped graphene nanosheets (NGNS) on carbon cloth	Carbon cloth	1 cm × 1.5 cm	597 m^2^/g	Pt	*E. coli*	1008 mW/m^2^	[109]
Carbon-based	Graphene oxide	Carbon paper	2 cm × 1cm	-	Ti	*S. oneidensis MR-1*	102 mW/m^2^	[90]
Carbon-based	3D-Graphene	Carbon cloth	0 mm × 5 mm (diameter × thickness)	9.41 m^2^	Pt	*E. coli*	1516 ± 87 mW/m^2^	[86]
Composites	Graphene/PPy	Carbon cloth	-	136 g/m^2^	Without catalyst	*S. oneidensis MR-1*	145 mW/m^2^	[110]
Carbon-based	Carbon cloth	Carbon cloth	-	6 cm^2^	Without catalyst	Wastewater	1292±69 mW/m^2^	[27]
Carbon-based	Glassy carbon	Carbon cloth	1.7cm × 1.8 cm	7 cm^2^	Pt	Anaerobic sludge	1905 mW/m^2^	[111]
Composite	Graphene powder/ Polytetrafluoroethylene on Carbon cloth	Carbon cloth	4 × 4 cm^2^	-	Pt	Anaerobic pre-treated sludge	0.329 mW/m^2^	[112]
Carbon-based	Carbon brush	Carbon cloth with gas diffusion layers	2.5 cm × 2.5 cm	16 cm^2^	Ti	Sludge	4.25 mW/m^2^	[113]
Carbon-based	r-GO sheets/ carbon cloth	carbon cloth	-	4.5 cm^2^	Pt	Anaerobic sludge	2.5 W/m^3^	[114]
Composite	TiO_2_ and r GO composite	Carbon fiber/brush	1 cm × 1 cm	Anode projected surface area 1 cm^2^	Ti	*S. putrefaciens CN32*	3169 mW/m^2^	[115]
Carbon-based	Graphite brush	Carbon cloth	3 cm × 2cm	8 cm^2^	Pt	Native wastewater	1280 mW/m^2^	[116]
Carbon-based	Carbon felt	Carbon fiber felt	2.5 × 2.5 cm	2.5 cm^2^	Pt	Anaerobic sludge	784 mW/m^2^	[117]
Composites	Polypyrrole/ graphene oxide	Carbon felt	3.0 cm × 2.0 cm × 0.5 cm	-	Pt	*S. oneidensis*	1326 mWm^−2^	[118]
Carbon-based + Polymer composite	RGO/ Carbon cloth-PANI	Carbon felt	1.8 cm × 1.8 cm	-	Pt	Anaerobic Sludge	1390 mWm^−2^	[119]
Composites	Graphene/Au composite	Carbon paper	-	6 cm^2^	Pt	*S. oneidensis MR-1*	508 mW/m^2^	[120]
Carbon-based	Graphene oxide wit CNT	Carbon cloth	-	-	Pt	*E. coli*	434 mWm^−2^	[121]
Carbon-based	Non-wet-proof carbon paper	Non-wet-proof carbon paper	-	10 cm^2^	Pt	Mixed community	188 mWm^−2^	[122]
Carbon-based	Carbon cloth /CNTs	Carbon cloth/CNTs	3cm × 6 cm	-	Pt	Domestic wastewater - acetate	65mW/m^2^	[123]
Carbon-based	Carbon paper	Carbon paper	2.5 cm × 4.5 cm	22.5 cm^2^	Pt	Primary clarifier overflow	600mW/m^2^ (anode area)	[7]
Composites	Graphite plates	Platinum meshes	-	155 cm^2^	-	*Shewanellaoneidensi*	1410 mW/m^2^	[124]
Carbon-based	Carbon mesh	Carbon mesh	7 cm^2^	-	Pt	Preacclimated bacteria from an active MFC	893 mW/m^2^	[125]
Carbon-based	Activated carbon cloth	Graphite foil	-	1.5 cm^2^ in projected area	Pt	D. desulfuricans strain	0.51 mW/cm^2^	[20]
Composites	Polypyrrole coating on carbon cloth	Granular activated carbon	Anode chamber: 450 mL, wet volume: 250 mL	-	Pt	Domestic wastewater	5 W/m^3^	[126]
Metal	Stainless steel	Stainless steel	20 × 30 cm,	0.12 m^2^	Pt	Marine sediments	23 mW/m^2^	[127]
Carbon-based	Non-wet proofed carbon cloth	Wet proofed carbon cloth	-	7 cm^2^	Pt	Domestic wastewater	766 mW/m^2^	[128]
Composites	Stainless Steel Mesh coated with Carbon cloth	Carbon black	-	7 cm^2^	Pt	Domestic wastewater	1610 ± 56 mW/m^2^	[129]
Carbon-based	Plain Carbon paper	Carbon paper	2.5 × 4.5 cm	-	Pt	Sediment sludge	33 mW/m^2^	[130]
Carbon-based	Granular graphite	Granular graphite	Granular diameters: 1.5–5 mm	817 m^2^	Pt	Mixture of sediment, aerobic and anaerobic sludge	8 W/m^3^	[131]
Carbon-based	Granular graphite	Graphite felts	40 mL	-	Pt	Mixture of sediment, aerobic and anaerobic sludge	83 ± 11 W/m3	[132]
Carbon-based	Graphite plate	Graphite fiber brushes	1.2 cm × 4.6 cm × 4 cm	28 cm^2^	Pt	Aerobic sludge	68.4 W/m^3^	[133]
Metal and metal oxide	Ti/TiO_2_	Pt meshes	-	-	Pt	Swamp sediments	2317 W/m^3^	[134]
Metal and metal oxide	Titanium rod	graphite felt	20 mm	20 ± 1 cm^2^	Pt	Pre-acclimated bacteria	-	[135]
Composite	Zero-dimension nitrogen-doped carbon dots modification with carbon paper	Carbon paper	2.5 cm^2^ × 2.5 cm^2^	-	Pt	*Pseudomonas*	0.32 mW/m^2^	[136]
Composites	Nickel foam/CNTs/PANI	carbon cloth	*-*	1 cm^2^ of anode surface-area	Without catalyst	*Shewanella Sp.*	113 W/m^3^	[137]
Metal and metal oxide	Titanium	-	2 cm × 2 cm	-	Pt	*G. sulfurreducens*	-	[138]

DMFC = Double Chamber microbial fuel cell; SMFC = Single chamber microbial fuel cell; Stainless Steel = SS. GO = Graphene oxide; PPy = Polypyrrole; CNT = Carbon nanotubes; Au = Gold; PANI = Polyaniline; Pt = Platinum.

**Table 3:** Effect of the anode on the performance of removal efficiency and energy production through MFCs.

Type of Pollutants	Electrodes		Target Analytes	Inoculation Source	Pollutant Removal (%)	Power Density	Reference
	Anode	Cathode					
Metal-based Water Pollutant	Graphite felt	Graphite plate	Cu^2+^	Anaerobic sludge	70	314 mW/m^3^	[158]
	Graphite plate	Graphite felt	CuSO_4_/CuO	Anaerobic sludge	>99	314 mW/m^3^	[159]
	Graphite felts	Graphite felts	Cr (VI)	Actinobacteria, Β-Proteobacteria,	5 mg/L with 93 25 mg/L with 61	-	[160]
	Carbon fiber felt	Carbon fiber felt	Cr (VI)	Anaerobic sludge	75.4 ± 1.9	970.2 ± 60.5 mW/m^2^	[142]
	Carbon fiber felt	Carbon fiber felt	V(V)	Anaerobic sludge	67.9 ± 3.1	970.2 ± 60.5 mW/m^2^	[142]
	Carbon brush	Carbon cloth	Ag^+^ ions	Sludge mixture	99.91	4.25 W/ m^2^	[142]
	Activated charcoal	Activated charcoal	Cr (VI)	Algae biomass	98	207 mW/m^2^	[161]
	Graphite felt	Graphite rod	Cr (VI)	*Shewanella* *oneidensis MR-1*	67	32.5 mW/m^2^	[162]
	Carbon cloth	Carbon cloth with Pt coating.	Oil sands tailings	Oil sands tailings affected water	97.8 Se, 96.8 Ba, 77.1 Mo, 32.5 Pb	392 mW/m^2^	[144]
	Carbon brush	Carbon cloth	Au^3+^	Tetrachloroaurate wastewater	99.89 ± 0.00	6.58 W/m2	[163]
	Carbon cloth	Graphite	Ag^+^	NH_3_ chelated silver waste water	99.9	317 mW/m^2^	[164]
	Graphite felt	Graphite felt	Co	Lithium cobalt oxide Solution	62.5 ± 1.8	298 ± 31 mW/m^3^	[165]
	Carbon cloth (no wet proofing)	carbon cloth (30% wet proofing)	Zn	Sewage sludge	90	3.6 W/m^2^	[166]
	Carbon fiber felt	Carbon fiber felt	V(V)	*Dysgonomonas and Klebsiella*	60.7	529 ± 12 mW /m^2^	[143]
	Carbon brush	Reduced Graphene oxide	Cu^2+^	*Geobacter and Pseudomonas,*	98	0.95 W /m^2^	[167]
	Carbon felt	Carbon felt	Cr (VI)	*Shewanelladecolorationis S12*, *K. pneumonia*	99.9	52.1 mW/cm^2^	[168]
	Graphite plate	Graphite plate	Platinum (Pt)	Anaerobic sludge bed	90	844.0 mW/ m^2^	[169]
Dyes-based Water Pollutant	Graphite rod	Graphite rod	Acid orange 7	Microbial consortium	78	0.31 ± 0.03 W/m^3^	[170]
	Granular graphite	Spectrographic pure graphite	Amaranth	-	82.59	137.37 mW/m^2^	[171]
	Plain carbon felts	Carbon felt	Congo red	Anaerobic sludge	86.4	400 mW/m^2^	[172]
	Graphite felt	Carbon paper	Congo red	Anaerobic sludge	70	72.4 mW/m^2^	[173]
	Activated Carbon	Hydrophobic carbon cloth	Model textile dyes	*Proteus hauseri*	75	103 mW/m^2^	[174]
	Porous carbon paper	Porous carbon paper	Active brilliant red X-3B	Aerobic sludges	90	213.93 mW/m^2^	[175]
	Plain carbon papers (non-wet proofed)	Carbon paper (wet-proofed)	Congo Red	Culture of aerobic and sludge	85	107 mW/m^2^	[176]
	Carbon cloth	Carbon cloth	Acid orange 7	*Shewanellaoneidensis*	>98	-	[177]
	Graphite-granules	Graphite-granules	Azo dye	Anaerobic sludge	85	34.77 mW/m^2^	[178]
	Activate carbon	Stainless steel mesh	Azo dye	Concentrated anaerobic sludge	96.5	0.852 W/m^3^,	[144]
	Graphite rods	Graphite rods	Acid navy blue R	Anaerobic sludge	-	0.125 mW/c m^2^	[179]
	Porous carbon cloth	Porous carbon cloth	Thionine-based textile Dyes	*Proteus hauseri*	50	83.4 mW/m^2^	[42]
	Unpolished graphite	Rutile– coated graphite cathode	Methyl orange	Anaerobic sludge	73.4	0.13 ± 0.03 mW/m^2^	[147]
	Carbon felt	Carbon felt	Azo dye	Mixed-culture sludge	94	8.67 mW/m^2^	[180]
